# Thymic Epithelial Cell Development and Its Dysfunction in Human Diseases

**DOI:** 10.1155/2014/206929

**Published:** 2014-02-03

**Authors:** Lina Sun, Hongran Li, Haiying Luo, Yong Zhao

**Affiliations:** Transplantation Biology Research Division, State Key Laboratory of Biomembrane and Membrane Biotechnology, Institute of Zoology, Chinese Academy of Sciences, Beichen Xi Road 1-5, Chaoyang District, Beijing 100101, China

## Abstract

Thymic epithelial cells (TECs) are the key components in thymic microenvironment for T cells development. TECs, composed of cortical and medullary TECs, are derived from a common bipotent progenitor and undergo a stepwise development controlled by multiple levels of signals to be functionally mature for supporting thymocyte development. Tumor necrosis factor receptor (TNFR) family members including the receptor activator for NF**κ**B (RANK), CD40, and lymphotoxin **β** receptor (LT**β**R) cooperatively control the thymic medullary microenvironment and self-tolerance establishment. In addition, fibroblast growth factors (FGFs), Wnt, and Notch signals are essential for establishment of functional thymic microenvironment. Transcription factors Foxn1 and autoimmune regulator (Aire) are powerful modulators of TEC development, differentiation, and self-tolerance. Dysfunction in thymic microenvironment including defects of TEC and thymocyte development would cause physiological disorders such as tumor, infectious diseases, and autoimmune diseases. In the present review, we will summarize our current understanding on TEC development and the underlying molecular signals pathways and the involvement of thymus dysfunction in human diseases.

## 1. Introduction

Thymus, as a primary lymphoid organ for T lymphocyte development and maturation, plays an essential role in keeping host cellular immune tolerance to self-antigens. Thymic epithelial cells (TECs) forming a 3-dimentional network critically shape T cell repertoire in thymus, though other antigen-presenting cells in the thymus were also involved [1]. Based on the location, TECs are divided into cortical TECs (cTECs) located in the outer cortex region and medullary TECs (mTECs) located in the inner medulla area, respectively. cTECs and mTECs play distinct roles in thymocyte positive and negative selections [1, 2]. Except for hyperplasia, thymomas, Nude syndrome, and thymic involution which occur in the thymus itself, the relationships of thymus dysfunction with other human diseases such as myasthenia gravis (MG), type 1 diabetes, and autoimmune diseases have been recognized [3, 4]. On the other hand, the thymus undergoes atrophy caused by several endogenous and exogenous factors such as aging, hormone fluctuations, and infectious agents, resulting in abnormal release of thymus-derived T cells and impaired host immunity [5]. In the present review, we will focus on our current understanding on TEC biology and the involvement of TEC dysfunction in human diseases.

## 2. Thymus Organogenesis and TEC Development

The rudimentary thymus arises from the endoderm of the third pharyngeal pouch around day 9 of embryonic development (E9) in mice. The thymic gland reaches its final anatomical location at about week 6 in the human fetus [6]. TECs are derived from nonhematopoietic cells which are negative for CD45 expression and positive for epithelial marker EpCAM. TECs are roughly divided into two groups—cTECs and mTECs, which are phenotypically and functionally different. cTECs and mTECs distinctively express different cytokeratin, in which most mTECs express cytokeratin 5 (K5) and K14 but low level of K8, whereas cTECs express K8 and K18 [7]. TECs that express both K5 and K8 (K5^+^K8^+^) are mainly located at the corticomedullary junction. They are part of cTECs or the immature progenitors for mTECs and cTECs. In addition, mTECs are positive for the expression of Ulex europaeus agglutinin-1 (UEA-1) on cell surface, but not Ly51 (UEA-1^+^Ly51^−^), while cTECs are UEA-1^−^Ly51^+^. With these markers, we can roughly distinguish mTECs and cTECs in immunofluorescence and flow cytometry assays. In addition, mature cTECs express high level of MHCII and protease *β*5t and thymus-specific serine protease (TSSP) participating in thymocyte positive selection, while mature mTECs express MHCII, CD80, autoimmune regulator (Aire) and tissue-restricted antigens (TRAs). Proteases Cathepsin-L and -S in mTECs mediate thymocyte negative selection. The major differences of cTECs and mTECs are briefly summarized in Table 1.

It has been reported that bipotent TEC precursors (TEPCs) could differentiate into both cTECs and mTECs [8–10]. The size of the TEC progenitor pool significantly controls the number of mature TECs and limits their recovery [11]. These TEPCs remained uncharacterized for some time. One recent study has shown that a group of TECs expressing cTEC marker CD205 represented TEPCs [12]. These progenitors first emerge as early as E11 when TECs just began Foxn1 expression, and they could generate both cTECs and Aire^+^ mTECs to establish a functional thymic microenvironment. In addition, the individual progenitors for cTECs and mTECs exist [13, 14]. mTECs highly expressing the tight-junction protein claudin-3 and claudin-4 (UEA-1^+^Cld3,4^hi^) may represent the progenitors specifically for Aire^+^ mTECs [14], while the progenitors for cTECs are phenotypically characterized as EpCAM^+^CD205^+^CD40^−^ [15].

**Figure 1:**
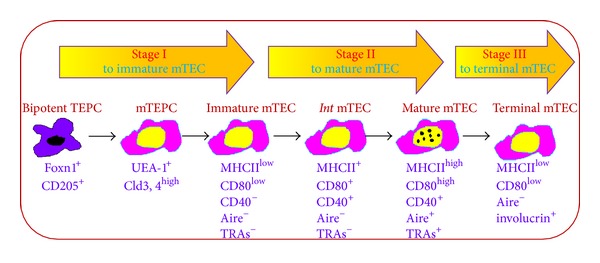
mTEC development stages and the relevant markers. The development of mTECs is roughly divided into 3 stages: CD205^+^ TEPCs first develop into progenitors specifically for mTECs characterized as high expression of claudin-3 and claudin-4 (UEA-1^+^Cld3,4^high^). mTEPCs develop into immature mTEC expressing UEA-1 but low level of MHCII and costimulatory molecules CD80, and CD40. As mTECs develop further into the middle mature stage, MHCII, CD80, and CD40 expression are upregulated but still without Aire and tissue-restricted antigens (TRAs) expression. The full mature mTECs highly express MHCII, CD80 and Aire (UEA-1^+^MHCII^high^CD80^high^Aire^+^) as well as upregulation of Aire-dependent and independent TRAs. Finally, mature mTECs enter into terminal differentiation stage as Aire^−^CD80^int/low^MHCII^low^ involucrin^+^ mTECs.

Generally, the development of mTECs is divided into 3 stages (Figure 1): bipotent TEPCs acquire mTEC sublineage differentiation orientation into immature mTECs expressing UEA-1 but low MHCII and costimulatory molecules CD80 and CD40. As mTECs develop into mature mTECs, MHCII CD80 and CD40 are upregulated concomitantly. mTECs in the middle mature stage do not express Aire and are functionally immature. The full mature mTECs are phenotypically characterized as high expression of MHCII and CD80 and Aire (UEA-1^+^MHCII^hi^CD80^hi^Aire^+^) as well as upregulation of Aire-dependent and Aire-independent TRAs participating in thymocyte negative selection [7]. Eventually, mature mTECs continue to develop into terminal differentiation stage by loss of CD80, MHCII, Aire, and TRAs expression, but with involucrin expression [16].

MHCII^hi^CD80^hi^Aire^+^ mTEC subset was previously considered to be the postmitotic end stage of mTECs which will be removed by apoptosis. However, accumulating evidence has shown that mTECs may continually develop beyond Aire^+^ stage. First, Aire^−/−^ mice have no Hassall's corpuscles (HCs) structure [44] which is formed from terminally differentiated epithelial cells. The presence of HCs follows the Aire^+^mTECs during ontogeny [27], and it seems that these mTECs are developed beyond Aire^+^ cell stage [45]. By using a cell fate-tracing method, Nishikawa and his colleagues demonstrated that Aire^+^CD80^hi^MHCII^hi^ mTECs developed into Aire^−^CD80^int⁡^MHCII^low^ end stage [16]. Recently, by using a transgenic mouse model in which LacZ reporter gene was under the control of Aire promoter, Wang et al. showed that a single mTEC had 2 to 3 weeks' life cycle, in which Aire was expressed only once within possible maximal 1-2 days [46]. The loss of Aire expression is accompanied by downregulation of MHCII, CD80 and TRAs. In the final developmental stage, mTECs lose their nuclei to become HCs and specifically express desmogleins (DGs) 1 and 3 [46]. So the expression of Aire, CD80, and MHCII undergoes dynamic changes from low to high to low expression eventually. The end stage of mTECs expresses involucrin, a marker of terminally differentiated epithelium. Consistently, the presence of involucrin^+^mTECs followed the Aire^+^mTECs during ontogeny [27].

In contrast to mTECs, the developing stages of cTECs remain poorly defined. It is proposed that TEPCs firstly develop into progenitors specific for cTECs (cTEPCs) phenotypically characterized as EpCAM^+^CD205^+^CD40^−^MHCII^−^. Unlike the common bipotent progenitors, cTEPCs could self-renew after thymus injury is recovered [47]. Concomitant with cTECs maturation, the expressions of CD40, MHCII, and a series of proteases participating in thymocyte positive selection are upregulated [15, 48–50]. *β*5t thymoproteasome in cTECs is required for MHCI-restricted CD8^+^ T cells production, while cathepsin-L and TSSP are important for MHCII-restricted CD4^+^ T cells generation. Clearly, it is required to investigate the specific markers for cTEPCs and cTEC subsets in different developing stages, which will significantly help us to study cTEC development and the relevant mechanisms.

## 3. Molecules Control TEC Development

TEC development is a complex and continuous process under control of extrinsic and intrinsic signal regulatory network. Tumor necrosis factor receptor (TNFR) family members including the receptor activator for NF*κ*B (RANK), CD40, and lymphotoxin *β* receptor (LT*β*R) are especially involved in determining mTEC formation and development, while fibroblast growth factor (FGF) and Wnt promote TEC expansion and functional maintenance. Transcription factors Foxn1 and Aire are essential for TEC development and functional maturation. The molecules involved in TEC development are summarized in Table 2.

### 3.1. The Effects of TNFR Family on TECs

It is highly recognized that TECs development and maturation are definitely dependent on their interaction with other cells in thymus such as thymocytes, fibroblasts, and mesenchymal cells. TNFR superfamily members and their ligands play an essential role in TECs especially mTECs development [51]. mTECs express a diverse set of TNFRs, and three of them including RANK, CD40, and LT*β*R have been proven to cooperatively control the thymic medullary microenvironment and self-tolerance establishment.

In the embryonic thymus, RANKL signals provided by CD4^+^CD3^−^ lymphoid tissue inducer (LTi) cells promote CD80^−^Aire^−^mTEC developing into CD80^+^Aire^+^mTECs [17]. Invariant V*γ*5^+^ dendritic epidermal T cells also made contribution to the development of Aire^+^ mTEC development through providing RANKL [18]. In the postnatal thymus, RANKL signal is provided mainly by positively selected CD4^+^ T cells [19, 20]. Disruption of the RANKL-RANK signaling in the postnatal thymus leads to reduction of mature UEA-1^+^CD80^hi^MHCII^hi^ mTECs. In contrast, mice deficient for osteoprotegerin (OPG, a decoy receptor for RANKL) developed thymic hyperplasia and had more mature mTECs [20]. Transplantation of RANKL^−/−^ thymus or transferring their splenocytes to immune deficient mice caused severe inflammatory cell infiltration and abundant production of autoimmune antibody [17, 19]. So the abnormality of RANKL-RANK signaling results in mTEC development arrest and the failure of T cells for self-tolerance.

CD40L-CD40 signaling pathway is also essential for mTEC development. CD40- or CD40L-deficient mice had obviously less mature mTECs and showed an autoimmune phenotype. Although the defects are less severe compared to RANK-deficient mice, CD40^−/−^RANKL^−/−^ double deficient mice displayed a greater reduction in mature mTECs and more severe autoimmune disease, implying that RANK and CD40 act cooperatively in modulation of thymic medullary microenvironment and self-tolerance [19–21]. In the postnatal thymus, CD40L signal provided by positively selected thymocytes (CD4^+^ and CD8^+^ T cells) promotes mTEC proliferation [22].

In the thymus, LT*β*R is mainly expressed on thymic stromal cells other than T and B lymphocytes. Two ligands for LT*β*R are discovered: LT*α*1*β*2 and LIGHT, in which the former consists of LT*α* and LT*β* subunits. The mature single positive thymocytes are the main source for LT*β*R ligands in the thymus [21]. Mice deficient in LT*β*R, its ligands, or downstream signal molecule nuclear factor-*κ*B-inducing kinase (Nik) caused defects of thymic medulla development including disorganized medullary architecture, significant reduction in overall mTECs, and retention of T cell maturation with autoimmune disease [23–25]. However, there is still controversy in the role of LT*β*R in Aire and TRAs expression. Previous work showed that lymphotoxin signaling is required for Aire and Aire-dependent as well as Aire-independent TRA expression [52]. The following research claimed that lymphotoxin signaling does not regulate Aire and TRAs expression in mTECs [53]. LT*α*- or LT*β*-deficient mice showed normal CD80, CD40, and Aire as well as TRAs expression despite reduced medulla area. The distribution of regulatory T cells (Tregs) and DCs in the thymus was also not affected [53, 54]. The inconsistent results regarding lymphotoxin signaling and Aire expression might be due to different TCR transgenic mouse models and the different detecting measures used in those studies [55], which need to be clarified in the future. One recent study showed that in embryonic mTEC development, the LT*β*R signal upregulated RANK expression in the thymic stroma, thereby promoting RANK signaling and mTEC differentiation [26]. Continued mTEC development into the involucrin^+^ stage also requires the activation of the LT*α*-LT*β*R signal provided by mature thymocytes [27]. Meanwhile, LT*β*R signals could indirectly influence mTEC development through regulating other stromal cells like MTS15^+^ fibroblasts which express the highest LT*β*Rl than TECs [54].

**Figure 2:**
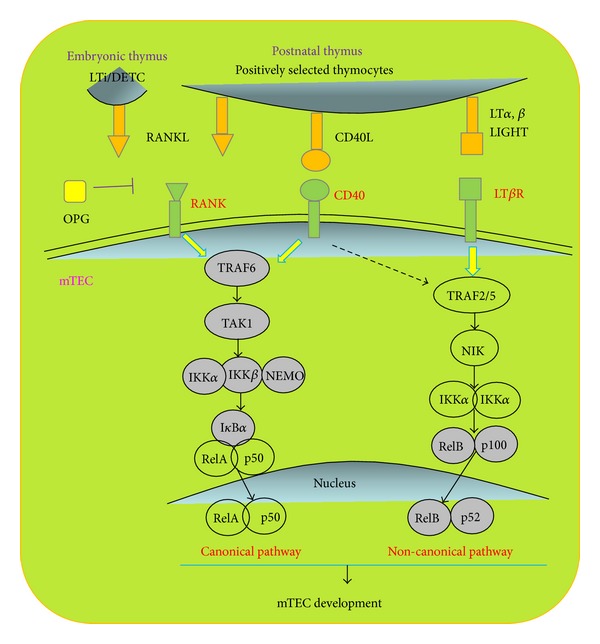
The effects and signaling pathways of TNFRs on TECs. Tumor necrosis factor receptor (TNFR) including the receptor activator for NF*κ*B (RANK), CD40, and lymphotoxin *β* receptor (LT*β*R) signalings is especially important for mTEC formation and development. In the embryonic thymus, RANKL is provided by CD4^+^CD3^−^ lymphoid tissue inducer (LTi) cells and Invariant V*γ*5^+^ dendritic epidermal T cells, while in the postnatal thymus, RANKL, CD40L, and LT*β*R ligands LT*α*, LT*β*, and LIGHT are provided exlusively by positively selected mature T cells. Canonical and noncanonical NF-*κ*B signal pathways are the major downstream of RANK, CD40, and LT*β*R. In classical NF-*κ*B pathways, TNFR-associated factor 6 (TRAF6) activates TGF-*β* activating kinase 1 (TAK1), which in turn activates the IKK complex composed of IKK*α*, IKK*β*, and NEMO. The IKK complex phosphorylates IkB*α* for degradation, leading to translocation of the RelA/p50 complex to the nucleus. Nonclassical NF-*κ*B pathways activate p52/RelB via TRAF2/5. IKK*α* is phosphorylated by NIK, which in turn triggers p100 partial degradation to p52 and then translocation to the nucleus together with RelB.

The signaling pathway downstream of RANK, CD40, and LT*β*R is usually NF-*κ*B signal [56]. In the thymus, NF*κ*B1 and RelA are mainly localized in cortical areas, whereas NF*κ*B2, c-Rel and RelB are in the medulla [57]. Both canonical and noncanonical NF-*κ*B signal pathways regulate mTEC development [25]. RANK and CD40 initiate activation of the classical NF-*κ*B pathways via TNFR-associated factor 6 (TRAF6). TRAF6-deficient mice showed severe destruction of medullary architecture and loss of UEA-1^+^mTECs [58]. In classical NF-*κ*B pathways, TRAF6 activates TGF-*β* activating kinase 1 (TAK1), which in turn activates the IKK complex composed of IKK*α*, IKK*β*, and NEMO. The IKK complex phosphorylates IkB*α* for degradation, leading to translocation of the RelA/p50 complex to the nucleus. In addition, RANK, CD40, and LT*β*R signaling could elicit nonclassical NF-*κ*B pathways via TRAF2/5 to activate p52/RelB [59]. IKK*α* is phosphorylated by NIK and in turn triggers p100 partial degradation to p52 and then translocation to the nucleus together with RelB. Mice deficient of genes in nonclassical NF-*κ*B pathways including NIK, IKK*α*, and RelB had abnormal thymus development with reduced UEA-1^+^ and/or Aire^+^ mTECs [60–64]. p52 deficiency results in less significant damage with little reduction in UEA-1^+^ and CD80^hi^ mTEC but with no obvious medullary architecture changes [65]. The effects and pathways of TNFRs on TECs are summarized in Figure 2.

### 3.2. The Effects of FGFs on TECs

FGFs boost thymopoiesis and promote differentiation by working on both thymocytes and TECs. FGF8 influences TECs indirectly by regulating neural crest cells (NCCs) survival and differentiation; therefore, FGF8 deficiency and NCCs deletion result in similar manifestation [28]. FGF7 and FGF10 conduct mainly as nutritional factors promoting TEC proliferation but not differentiation. Loss of FGF10 causes defects of thymus development and alters thymic cytokeratin expression pattern [29]. Development of thymus in mice deficient of FGF receptor R2-IIIb (FGFR2IIIb), receptor for FGF7 and FGF10, is blocked at E12.5 when TECs just emerge. However, FGF signal is not always enhancing TECs. When thymus and parathyroid glands are derived from the third pharyngeal pouch endoderm, localized inhibition of FGF signaling is essential for normal *Gcm2*, *Bmp4* and *Foxn1* expression and thymus/parathyroid detachment [66].

FGF7 is known as keratinocyte growth factor (KGF). Mature CD4^+^ and CD8^+^ thymocytes and fibroblasts are the main source for KGF in the thymus. KGF acts on both thymocytes and TECs, promoting their proliferation and function [30]. Applying KGF into *RAG*-deficient mice increased medullary compartment [31]. Administration of KGF protects the thymus against damage from radiation or graft-versus-host disease thus enhancing immune reconstitution after hematopoietic stem cell transplantation [32–34]. KGF attenuates thymic aging in elderly individuals, protects medullary architecture, and promotes T cell production [35, 36]. KGF regulates a series of genes associated with TEC function and T cell development including *BMP2*, *BMP4*, *Wnt5b,* and *Wnt10b *via activation of p53 and NF-*κ*B signal pathway [30].

### 3.3. The Effects of Wnt and Notch on TECs

Wnt receptors are exclusively expressed on TECs and Wnt regulates Foxn1 expression in the thymus [37]. Wnt4 is predominantly produced by TECs including both mTECs and cTECs [38]. Wnt4 controls thymopoiesis and thymus size by regulating TEC, thymocyte, and their progenitor proliferation [38, 39]. Wnt4 protects TECs from dexamethasone-induced injury [40]. Overexpression of DKK1, an inhibitor of Wnt4 in TECs, leads to thymic atrophy, reduction of TEPCs, and decreased TEC proliferation, features similar to thymic aging [67]. Therefore, Wnt4 becomes an indication for thymic senescence [68]. With ageing, the expression of Wnt4 and its downstream target Foxn1 is downregulated. On the other hand, one of the Wnt4 target gene, connective tissue growth factor, is involved in a negative feed-back loop suppressing Wnt expression, which is important for initiation of thymic senescence [69]. Thus, Wnt plays an important role in the thymic aging.

Development of both TECs relies on cell-cell interactions between the developing T-lymphocytes and the thymic epithelium. Such interdependency between thymocytes and TECs is often referred to as “thymic crosstalk.” Notch signaling represents one important molecular example for thymic crosstalk. In the thymus, both TECs and thymocytes express various Notch receptors and their ligands [70]. It is widely accepted that Notch ligands expressed on TECs are essential for T cell lineage commitment and maturation [71–73]. Further studies focused on the opposite direction of the crosstalk in which Notch activation played an essential role in TEC development. Jagged and Delta proteins are the main ligands for Notch receptor. Jagged2 gene mutant mice display defects in thymic morphology and impaired differentiation of *γδ*T cells [41]. In fetal thymic organ culture system, B cells enforced to express Delta-like-1 could efficiently induce TECs development to establish three-dimensional architecture of thymic environment [42]. However, overactivation of Notch signaling also causes regression of the thymus. Targeted expression of Jagged1 in the thymocyte progenitors leads to thymic atrophy by induction of apoptosis of TECs [43]. Accordingly, an increase in Notch and Delta expression in aging thymus was noticed [74]. The molecule regulating networks involved in the role of Notch in TECs need to be studied.

### 3.4. The Effects of Foxn1 on TECs

Transcription factor Foxn1 plays a crucial role in TEC development. Mice deficient in Foxn1 (Foxn1^nu/nu^, nude mice) have atrophic thymus and few T cells in the periphery, leading to severe immune deficiency [75]. Foxn1 expression was first detectable on E11.25 in mice, the stage between thymus anlage formation and TEC development [88]. Foxn1, expressing on almost all TECs, regulates mTEC and cTEC differentiation and function in the fetal and adult thymus [78]. In Foxn1^nu/nu^ mice, the earliest stage of TEC development was not impaired, in which the common progenitors could persist even in the postnatal thymus. However, thymus development was arrested after initial formation of the organ anlage (about E12.0 in mice) without hematopoietic precursor colonization [9, 76]. Foxn1^Δ/Δ^ mice with a hypomorphic Foxn1 allele, lacking exon 3 of Foxn1, had a highly cystic thymus, containing no discernible cortical or medullary regions [79]. In mouse models with conditional deletion of Foxn1, ubiquitous deletion of Foxn1 after birth, caused dramatic thymic atrophy in 5 days with more severe defects in mTECs (especially the MHCII^hi^UEA-1^hi^ mature population) than cTECs [80]. It was demonstrated that aging-related loss of Foxn1 caused thymic epithelial cysts in medulla and perturbed negative selection [81]. Recently, it is demonstrated that Foxn1 is required for stable entry into both the cortical and medullary TEC development lineage in Foxn1 dosage-dependent manner [77]. Overexpression of Foxn1 attenuated age-induced thymic involution. In old *Foxn1* transgenic mice, age-associated thymic atrophy was diminished, and the total number of EpCAM^+^ and MHCII^hi^ TECs was higher [82]. The accumulated studies collectively suggest that Foxn1 is a powerful regulator of TEC development on multiple stages and respects (Table 3): (1) Foxn1 is dispensable for earliest progenitors (TEPCs) presence [75, 77]; (2) Foxn1 is required for the differentiation from TEPCs to cTEC and mTEC sublineages; (3) Foxn1 participates in TEC proliferation [83, 84] and terminal differentiation [77, 85, 86]; (4) Foxn1 regulates the differentiation of TEC sublineages in postnatal thymus and aging. In addition to the function in regulating TEC development, Foxn1 also contributes to the vascularization of the murine thymus. In the nude thymus, CD31^+^ endothelial cells are not detected in the epithelial region [87], indicating that Foxn1 may indirectly regulate TEC and thymocyte development via controlling thymic vascularization.

**Figure 3:**
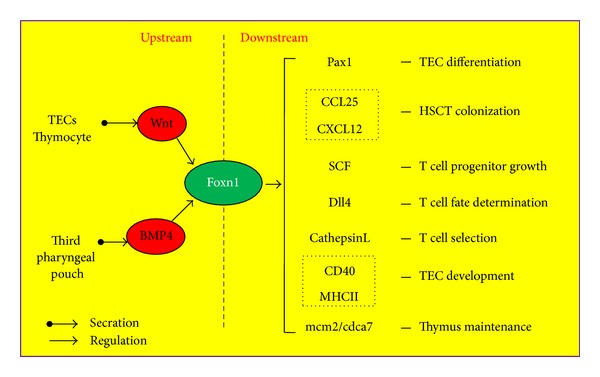
The molecular regulating network of Foxn1 in TECs. Foxn1 in TECs regulates a series of genes involved in the thymus development and function, and the expression of Foxn1 itself is strictly under control. Wingless (Wnt), provided by TECs and thymocytes, positively regulate Foxn1 expression in TECs. Bone morphogenic proteins (BMPs) expressed in the third pharyngeal pouch modulate Foxn1 expression during fetal thymopoiesis. Genes are regulated by Foxn1 in TECs are determined so far: Pax1, regulating TEC differentiation and proliferation during thymopoiesis; CCL25 and CXCL12, for hematopoietic stem cell localization in the thymus; SCF, Dll4 and CathepsinL, participating in T cell development and selection; CD40 and MHCII, involved in TEC development and function.

Foxn1 directly or indirectly regulates a series of genes involved in diverse aspects of thymus development and function [77]. Meanwhile, the expression and maintenance of *Foxn1* gene in thymus are strictly under control [77]. The regulation network of upstream and downstream Foxn1 is briefly summarized in Figure 3. *Pax1*, expressed on the third pharyngeal pouch at E9.5 and essentially regulating TECs differentiation and proliferation, is Foxn1-dependent [89]. *CCL25* and *CXCL12*, modulating hematopoietic stem cell localization in the thymus and stem cell factor (SCF), promoting T cell progenitor growth, were undetectable in Foxn1-deficient thymus [90–92]. Foxn1 deficiency also caused diminishment of Delta-like-4, ligand for Notch which controls hematopoietic stem cells specifically differentiated into early T cell progenitors [91, 93]. In addition, *CathepsinL*, *CD40*, and *MHCII *involved in TEC development and function are regulated by Foxn1 directly or indirectly [77]. Importantly, it is identified that Foxn1 regulates development of TECs and thymocytes through mcm2/cdca7 axis in zebrafish thymus [92].

Wnt and bone morphogenic proteins (BMPs) are two main regulators upstream of *Foxn1* gene. In the thymus, mostly Wnt4 and Wnt5b, produced by TECs and thymocytes, regulate *Foxn1* expression in TECs through both autocrine and paracrine manners [37]. Overexpression of Noggin, an antagonist of BMP4 in TECs, leads to atrophic thymus and small number of thymocytes [94]. In the fetal thymic organ culture, BMP4 promotes Foxn1 expression on TECs and thereby improving thymic microenvironment for thymopoiesis [95].

### 3.5. The Effects of Aire on TECs

Aire is not only a marker for mature mTECs but also regulates mTEC development and differentiation (reviewed in [96]). Aire-deficient mice showed morphological changes in medullary components with decreased mTECs [44]. It is demonstrated that the numbers of mTECs expressing involucrin, a marker for terminal differentiated epithelium, were reduced in the Aire-deficient thymus [20]. The most important function of Aire is regulating expression of a panel of peripheral self-antigens in mTECs and promotes the antigen presentation ability of mTECs, participating in T cell negative selection and selftolerance establishment [97]. The mRNA levels of Aire in mTECs could also determine the expression of peripheral tissue antigen genes [98]. Aire deficiency caused a severe autoimmune disease manifestation with inflammatory cell infiltration in multiple organs and autoimmune antibody production [99, 100]. So far, three main manners are proposed for Aire regulating TRAs expression [101], which are summarized in Table 4: (1) Aire as a classical transcription factor directly initiates transcription of target genes; (2) Aire increases TRAs expression nonspecifically by loosening up the chromatin structure; (3) Aire functions through epigenetic modification. Aire could recognize epigenetic site of unmethylated histone 3. Following demethylation, Aire enhances target gene transcription via either itself directly or recruiting other transcriptional activators indirectly.

Recent reports indicated that Aire also controlled the expression of microRNAs in mTECs, which in turn play a crucial role in maintaining thymic microenvironments [102, 103]. Giraud and colleagues found that Aire could induce transcription of target genes by unleashing stalled RNA polymerase in mTECs [104]. In addition, an increase of mTEC expressing truncated Aire protein was observed in Aire-deficient thymus, indicating that these mTECs would be eliminated in wild-type thymus and shed light on Aire's proapoptotic activity [105]. Overexpression of Aire in an mTEC cell line caused overt apoptosis [106]. The mechanism of this proapoptotic activity is in part associated with nuclear translocation of stress sensor and proapoptotic protein GAPDH [107].

## 4. Thymic Dysfunction and Human Diseases

The major biological function of the thymus is to generate a diverse repertoire of T cells to constitute an important part in host adaptive immune system against foreign pathogens, while thymus also plays a critical role in self-tolerance via thymic negative selection and the production of Treg cells. TECs are the most important components in thymic microenvironment supporting thymocyte development and self-tolerance establishment. Since the thymus plays a key role in keeping balance between host immunity and tolerance, it is obvious that thymic dysfunction causes a diversity of diseases in humans (Table 5).

### 4.1. Thymus Tumors

Thymus tumors are scarce. Thymomas and thymic carcinomas are two major epithelial tumors of the thymus. Thymomas are neoplasms arising from TECs, usually with organotypic features (have normal thymus), numerous maturing thymocytes, and autoimmune syndromes such as myasthenia gravis (MG). Thymic carcinomas are malignant epithelial tumors with invariability and invasiveness and without organotypic feature and autoimmune disease [108].

### 4.2. Diseases Related to Immune Deficiency

Abnormality of the thymus is always concomitant with lower production of functional T cells and leads to immunodeficiency. Immunodeficiency in hosts means higher susceptibility to pathogens infection including viruses, bacteria, and protozoa, as well as decreases in antitumor immunity. Abnormalities in TEC development lead to dysfunction of T cells which could cause chronic inflammatory disease. Targeted gp39 (CD40L) overexpression in thymocytes caused loss of cTECs and mTEC expansion, with decline in thymocyte numbers and morphologic features of chronic inflammatory bowel disease (IBD) [109].

### 4.3. Autoimmune Diseases

Central self-tolerance is established in the thymus by at least two main mechanisms: (1) negative selection—clonal deletion of self-antigen reactive T cells (2) generation of self-antigen-specific natural regulatory T cells (nTregs) to downregulate immune response. Impairment or breakdown of the thymic self-tolerance plays a primary role in the development of some autoimmune diseases. More and more evidence showed the correlation between thymus dysfunction in self-tolerance and autoimmune diseases.

In humans, *Aire* mutation results in autoimmune polyendocrinopathy-candidiasis-ectodermal dystrophy (APECED), also known as autoimmune polyendocrinopathy syndrome type 1 (APS-1). APECED is a rare systemic autoimmune disease characterized by chronic mucocutaneous candidiasis, hypoparathyroidism, and adrenal insufficiency. Different from many other autoimmune diseases, APECED is caused by a single gene mutation [110]. APECED is the first time for us to find the important autoimmune regulator Aire [111]. In mature mTECs, Aire drives organ-specific antigens expression on mTECs and mediates negative selection of autoreactive T cells. Therefore, failure of central tolerance based on tissue-restricted antigens expression could result in a series of autoimmune diseases in multiple organs.

Myasthenia gravis (MG) is a neuromuscular autoimmune disease characterized as muscle weakness and fatigability caused by T cell-dependent autoantibodies against neuromuscular junction. In MG patients, autoantibodies could directly attack muscle acetylcholine receptors (AChR), muscle-specific receptor tyrosine kinases (MuSK), and even muscles themselves. The exact trigger of MG is unclear; however, it is certain that alteration of the thymus and TECs is involved in MG pathogenesis. TEC dysfunction contributes to MG pathogenesis in several ways: defects in negative selection by producing AChR-reactive CD4^+^ T cells; overexpression of various cytokines and chemokines to recruit peripheral lymphocytes to the thymus leading to thymic hyperplasia, a hallmark of MG [112, 113].

Type 1 diabetes (T1D) is an autoimmune disease resulting from destruction of pancreatic islet *β* cells. It is widely accepted that the absence or failure of immune tolerance to islet *β* cells is the primary cause for development of T1D. Previous results have demonstrated that all the members of insulin gene family were expressed in mTECs [114]. Insulin1 and insulin2 are two Aire-dependent TRAs expressed in mTECs. Decreased expression of T1D-related antigens in the thymus or Aire deficiency would break down the self-tolerance to islet *β* cells leading to the development of T1D [115]. Other autoimmune diseases related to abnormalities of self-tolerance by organ-specific antigens expression on mTECs are autoimmune thyroiditis, rheumatoid arthritis, multiple sclerosis (MS), autoimmune myocarditis, Graves' disease, and so forth.

CD4^+^CD25^+^Foxp3^+^ nTreg cells are developed in the thymus as negative regulation candidate to control peripheral self-tolerance. Dysfunction of the negative regulatory system mediated by nTreg cells could also play a crucial role in the development of autoimmune diseases. Loss of CD4^+^CD25^+^Foxp3^+^ nTreg cells alone is sufficient to induce autoimmune reaction. In humans, mutation of FOXP3, a specific transcription factor for nTreg cells, will cause a failure of nTreg cell development and will subsequently cause X-linked immunodeficiency syndrome IPEX (X-linked syndrome, immune abnormality, polyendocrinopathy, enteropathy) [116]. Dysfunction of nTreg cells means loss of balance between CD4^+^ T helper cells subsets (Th1, Th2, Th17, Treg) which is supposed to participate in other autoimmune diseases, such as MG [117] and T1D [115]. It is reported that a direct role for CD4^+^CD25^+^ Treg cells in restraining B cell autoantibody production and defects in CD4^+^CD25^+^ Treg cells may be crucial to the development of primary biliary cirrhosis [118]. Defective thymic selection with higher Th1 response and lower nTreg cells numbers spontaneously develops IBD-like colitis, suggesting that the impaired control of self-reactive T cells by nTreg cells could result in autoimmune diseases [119].

In conclusion, thymus (TECs) dysfunction participates in autoimmune disease development mainly through the abnormality in the following two aspects: (1) self-tolerance established by Aire-mediated tissue-restricted antigens expression on mTECs; (2) negative regulatory system formed by CD4^+^CD25^+^Foxp3^+^ nTreg cells.

### 4.4. Thymus Defects Caused by Diseases

More and more observations have implied that thymus is very sensitive and fragile to many physiological disorders such as infection, autoimmune diseases, and aging [120]. A variety of infectious agents such as viruses, bacteria, and protozoa would cause thymic atrophy characterized largely by the depletion of thymocytes (especially CD4^+^CD8^+^ T cells). Thymic microenvironment of epithelial network is also affected by loss of mTECs and cTECs compartment, accumulation in extracellular matrix deposition. In HIV-infected children and adults, thymic dysfunction and involution including thymocyte apoptosis and severe TEC damage occur during disease progression [121, 122]. Thymic disorders are very common in some autoimmune diseases. Systemic sclerosis (SSc) is a connective tissue autoimmune disease related to self-tolerance failure. Thymus hyperplasia is present in a significant number of SSc patients. Moreover, in advanced SSc patients, thymus involution occurred [123]. In addition, thymic hyperplasia is commonly observed in Graves' disease [124] and MG [113].

Thymus involution remains a significant marker for senescence. In aged mice or humans, thymus size is reduced, as well as TECs, thymocytes, and peripheral T cells. Aged thymus has disorganized thymic architecture, increased cavity and cysts, and more fibroblast and fatty cells [74, 125, 126]. Besides, the thymus is sensitive to malnutrition. Protein energy, vitamin, trace element, and Zn^2+^ deficiencies could cause multiple thymic defects including thymic atrophy [127]. Accordingly, thymus defects lead to lower functional T cells production and self-tolerance breakdown, which could in turn exacerbate the disease progression. Therefore, thymus has been proven extremely important in maintenance of host immunity and self-tolerance and protection from the occurrence and progression of many diseases and aging.

## 5. Concluding Remarks

The fundamental function of thymus is to establish host immunity with self-tolerance. TECs are the most important components in thymic microenvironment supporting thymocytes development and directing central tolerance. Multiple signals and cellular interactions are required for the maturation, expansion, and maintenance of thymic epithelial compartments. TNFR signals including RANKL, CD40L, and lymphotoxin cooperatively control the thymic medullary microenvironment and self-tolerance establishment, while FGFs, Wnt, and Notch signals are essential for TEC and thymocyte expansion and functional maintenance. Foxn1 is a powerful modulator of TECs lineage progression in fetal and adult thymus in a dose-dependent manner. Aire expression in mature mTECs drives mTEC development and directs self-tolerance establishment. Thymic dysfunction is associated with many diseases including tumors, infectious diseases, and autoimmune diseases. On the other hand, the thymus is a primary target organ for many physiological disorders such as pathogen infections, autoimmune diseases, aging, and malnutrition. Since the thymus plays an important role in immunity and diseases, understanding the mechanisms for TEC differentiation and function would offer the possibility for the clinical application of “modification of thymus function” to improve our cellular immunity in physiological and pathological conditions such as infections, autoimmune diseases, and aging.

## Figures and Tables

**Table 1:** The major differences between cTECs and mTECs.

	cTECs	mTECs
Location	Cortex	Medulla
Cytokeratin expression	K8, K18	K5, K14
Surface marker	Ly51, CD205	UEA-1, CD80
Maturation	MHCII^hi^, *β*5t	MHCII^hi^, CD80^hi^, Aire, TRAs
Proteases	*β*5t, Cathepsin-L, TSSP	IFN-*γ*-induced *β*5i, *β*1i, *β*2i
		Cathepsin-L, S
T cell selection	Positive	Negative

^hi^High expression; TSSP: thymus-specific serine protease; TRAs: tissue restricted antigens.

**Table 2:** Molecules involved in TEC development.

Family	Molecule	Receptor	Source	Function	References
TNF	RANKL	RANK	Embryonic: LTi, DETC	Thymic medulla formation	[17, 18]
			Postnatal: positively selected thymocytes	mTEC development	[19, 20]
	CD40L	CD40	Positively selected thymocytes	mTEC development	[19–22]
				mTEC proliferation	[19–22]
	LT*α*1*β*2, LIGHT	LT*β*R	Positively selected thymocytes	mTEC development	[23–25]
				Promote RANK signals	[26]
				mTEC terminal differentiation	[27]

FGFs	FGF8		Pharyngeal region	Thymopoiesis	[28]
	FGF10	FGFR2IIIb	Positively selected thymocytes	mTEC proliferation	[29]
	FGF7	FGFR2IIIb	Positively selected thymocytes	mTEC and thymocyte Proliferation	[30, 31]
				Protect thymus damage	[32–34]
				Enhance thymopoiesis	[35, 36]

Wnt	wnt4	Frizzled	TECs, fibroblast	Regulate Foxn1 expression	[37]
				Thymopoiesis	[38–40]

Notch	Jagged, Delta	Notch	Thymocyte progenitor	TEC survival and development	[41–43]

DETC: invariant V*γ*5^+^ dendritic epidermal T cells; RA: retinoic acid.

**Table 3:** Foxn1 regulates multiple TEC development stages.

Capability	Regulation aspects	References
Dispensable	TEPCs appearance	[75–77]
	TEC fate-choice	[77]
	Medullary sublineage divergence	[77]

Indispensable	TEPCs into cTEC and mTEC sub-lineage	[77]
	TECs differentiation	[76–82]
	TECs proliferation	[83, 84]
	TECs termination	[77, 85, 86]
	Thymic vascularization	[87]

**Table 4:** Models of Aire regulate TRAs expression.

Model	Classical transcription factor	Random transcriptional activator	Epigenetic tag recognition
Manner	Bind to promoter of target genes initiating the transcription of master transcription factors	Loosening the chromatin structure	Bind modified histones

Evidence	DNA binding domain activating transcription Recruit transcriptional molecules	DNA accessibility function DNA sequences recognition	Demethylation histone 3 interact with transcription cofactors

**Table 5:** Thymic dysfunction and human diseases.

Level	Thymic dysfunction	Immunity	Disease
Molecular	Aire gene mutation	Autoimmunity	APECED
	Foxp3 gene mutation	Autoimmunity	IPEX

Cellular	Thymic epithelial tumor	Deficiency/autoimmunity	Thymomas
	Thymic epithelial tumor	Deficiency	Thymic carcinoma
	Treg dysfunction	Autoimmunity	IBD

Individual	Thymus infection/injury	Deficiency	*Infectious diseases: *
			AIDS (HIV)
			measles (measles virus)
			Ebola infection (Ebola virus)
			syphilis (bacteria)
	Absence of self-tolerance	Autoimmunity	*Autoimmune diseases: *
			myasthenia gravis
			type 1 diabetes
			autoimmune thyroiditis
			rheumatoid arthritis
			multiple sclerosis
			autoimmune myocarditis
			Graves' disease
